# Special Issue on E-Health Services

**DOI:** 10.3390/ijerph17082885

**Published:** 2020-04-22

**Authors:** Rolf Wynn, Elia Gabarron, Jan-Are K. Johnsen, Vicente Traver

**Affiliations:** 1 Department of Clinical Medicine, Faculty of Health Sciences, UiT–The Arctic University of Norway, 9037 Tromsø, Norway; 2 Norwegian Center for E-Health Research, University Hospital of North Norway, 9038 Tromsø, Norway; elia.gabarron@ehealthresearch.no; 3 Department of Clinical Dentistry, UiT The Arctic University of Norway, 9037 Tromsø, Norway; jan.a.johnsen@uit.no; 4 SABIEN-ITACA, Universitat Politècnica de València, 46022 València, Spain; vtraver@itaca.upv.es

**Keywords:** e-health services, telemedicine, social media and health, health

## Abstract

The importance of e-health to citizens, patients, health providers, governments, and other stakeholders is rapidly increasing. E-health services have a range of advantages. For instance, e-health may improve access to services, reduce costs, and improve self-management. E-health may allow previously underserved populations to gain access to services. Services utilizing apps, social media, or online video are rapidly gaining ground in most countries. In this special issue, we present a range of up-to-date studies from around the world, providing important insights into central topics relating to e-health services.

## 1. Introduction

Increasing and aging populations with more chronic illnesses are straining health services in both developed and developing countries. In this situation, the prevention of disease and the encouragement of healthy lifestyles becomes even more important. A shift of this kind necessitates more active patient engagement and patient involvement in health care, and e-health has to play a central part in this process. E-health services may be easier to access than traditional services in remote and rural areas and reduce the time spent by users on travel and appointments. It may be easier to offer the services to many people at a low cost. Thus, e-health services may improve the immediacy of access as well as the equality of access to quality health information and improve self-management and thereby help to alleviate the burden on health services. In addition, e-health can improve the quality of health services by increasing shared decision-making and by empowering citizens, patients, and health care professionals [1,2].

E-health can be defined as the use of information and communication technology for the enablement or improvement of health care [3]. Rapid technological development with increasing internet access around the world and the pervasiveness of smartphones makes e-health relevant to all. The growing coverage of mobile phones in low- and middle-income countries is allowing access to health information and other e-health services to people in underserved areas [4,5,6].

E-health has expanded from web-based services to mobile health apps, online video services, and social media, and new services and technologies are constantly being presented. A few examples of e-health services that are already in use in many countries around the world are online consultations, electronic patient records, digital radiological systems, decision-support tools, self-help apps, telemonitoring, and e-prescriptions.

The most frequently used e-health service, by far, is online health information, and studies from Europe and the US suggest that more than half of the general population and most internet users have used the internet to search for information about health and illness [2,7,8,9]. Of those who do search for health information online, about 6 in 10 take some type of action based on the information they find online [2,8]. While the access to e-health information and other services is increasing around the world, there still remains a divide between those who use and those who do not use these digital tools. This divide is linked to several factors, including, not least, socio-economic differences [2,9].

While most online health information searching seems to start at a search engine, social media such as Facebook and Twitter and online video services such as YouTube are likely to play an increasingly important role as sources of health information in the future [10,11]. Patients will be engaged in participating in their health care through a range of applications, including social media and mobile apps [12]. While social media now seem often to be used to provide information and support, mobile apps seem to be popular, especially for lifestyle issues such as exercise and dieting and for the self-monitoring of other health and illness variables. However, while the applications are beneficial to most, they may also, in some cases, worsen people’s health by spreading misinformation or encouraging eating disorder behaviors or self-harm [4,13,14,15]. In any case, as it stands today, many health care services do not seem to be fully utilizing the potential of these media that are rapidly gaining ground. Therefore, more research needs to be done on how to move from innovation into adoption in daily practice.

## 2. The Special Issue

In this special issue of the IJERPH on e-health services, we have included papers that cover a wide repertoire of services and methodological approaches, especially from medical, psychological, and societal perspectives. While the extent of e-health services is too big to be covered in full in this special issue, our included papers provide insight into several central topics within the field of e-health. We believe we have created a special issue that will provide readers with up-to-date insights into e-health services from around the world that would help researchers and other stakeholders to shape a better future.

Del Hoyo and collaborators [16] describe a study where they worked to adapt the TECCU telemonitoring app to IBD-patients’ needs and preferences. Drawing on a qualitative methodology involving successive focus groups, they identified three main themes that were central to the discussions: platform usability, the communication process, and the platform content. The app was valued for its usability and personalized monitoring. Through the study, further improvements were made to the app’s messaging system, and educational content was continuously updated.

In their paper on e-health communities for doctors, Li and collaborators [17] study the interaction of 102 doctors in the “Lilac Forum”. They found that the frequency of interaction between the participants varied due to factors such as differences in their professional standing (titles) and differences in degree of participation.

The paper from Misawa and co-authors [18] describes a case where the rate of Japanese citizens receiving colorectal cancer examinations had significantly increased due to the application of machine learning and nudge theory. In this study, machine learning—based on historical data from designated periodical health examinations, digitalized medical insurance receipts, and medical examination records for colorectal cancer—was used to deduce segments to whom receiving the examination was recommended. As a result, 3264 (26.8%) out of the 12,162 recommended subjects received the examination, which exceeded the upper end of the initial plan (19.0%).

The erroneous use and overuse of antibiotics has led to problems of bacterial resistance to antibiotics, increases health care costs, and gives patients unnecessary side-effects. E-health systems such as the Rational Antibiotic Use System (RAUS) may help with information, prescription support, and the monitoring of antibiotic usage. Shanshan Guo et al. [19] explore the impact of the RAUS on a large Chinese hospital. The findings suggest that the implementation of the system did not result in financial losses to the hospital, although the prescription of antibiotics was reduced—thereby providing encouragement for other hospitals to implement programs to reduce antibiotic prescribing.

In their paper on the “COPD-Life” program, Charlotte Simonÿ and coauthors [20] describe the program’s rationale and content. The program for COPD patients was delivered as a study intervention by an interprofessional team of clinicians collaborating from both the hospital and the municipal health care system in Denmark. Making use of two-way audio and visual communication software, 15 patients participated in the intervention via a tablet computer from their private setting. The intervention contained elements of instruction, conversation and exercise and aimed to draw on e-health to empower the patients to take better care of themselves.

In their paper on quality of life in patients following pacemaker implantation in a Norwegian hospital, Remedios López-Liria et al. [21] compare follow up through standard outpatient visits to follow up through remote monitoring. While the health-related quality of life was slightly better after 12 months in the group that received standard follow up, the difference was not statistically significant. Moreover, the frequencies of emergency visits and re-hospitalizations did not differ between the groups, suggesting that remote follow up should be further explored as an option for this group of patients.

In an analysis drawing on structural equation modeling, Yuan Tang and co-authors [22] examine which factors are of importance to patients’ acceptance of online medical websites in China. Based on their analyses, they propose a modified technology acceptance model and conclude that there is a need to further improve trust and to reduce the perceived risk to users in order to increase acceptance of the service.

There is a growing trend of individuals seeking health information on social media. Health authorities could benefit from these media to disseminate validated health information. By identifying engaging factors to their social media posts, they could further enhance the impact of the information. In an observational study by Afiq Izzudin A. Rahim et al. [23], the factors associated with engagement rates on the Facebook page of the Ministry of Health of Malaysia were analyzed. They found that only 39% of the posts by the Ministry of Health had good engagement rates. The posts that were the most successful were typically on the topics of health education or risk communication, included a video, and were posted in the afternoon or after office hours. The authors’ findings imply that taking these factors into consideration when posting on social media could further improve engagement rates and thereby the successful dissemination of important health-related information to the public.

In their paper, Sabina Asensio-Cuesta and co-authors [24] describe the development and assessment of an app for cell phones. The purpose of the app was to monitor the physical, psychological, social, and environmental aspects of patients receiving cancer treatment to indicate their quality of life (QoL). The authors tested the app in a pilot study with university volunteers from Spain and concluded that they could verify the plausibility of detecting human activity indicators directly related to QoL.

Kolasa and collaborators [25] performed a systematic literature review of assessment guidelines for digital health interventions. In the 11 identified guidelines, safety, clinical effectiveness, usability, economic aspects, and interoperability were most often discussed. Based on the review, the authors present important recommendations, including on methodology.

In a review, Almeida and collaborators [26] examine studies that assess the usability of pain-related apps. A main finding was that a majority of the studies did not use valid instruments or a triangulation of methods to assess usability. Drawing on their findings, the authors present recommendations for future studies in the field.

In their review of which sociodemographic factors influence the use of e-health in individuals affected by chronic conditions, Fabienne Reiners and co-authors [27] find that e-health seems to be the least used by the individuals that might need it the most, such as older individuals affected by chronic diseases, with low incomes and low educational levels, living in rural areas. Drawing on their review findings, the authors recommend tailoring the delivery of e-health services to address the inequality in the use of e-health, for instance, by using different ways of delivering the information or using different devices.

In a study protocol, Anish Menon and co-authors [28] describe a pilot randomized controlled trial (RCT) for a mobile diabetes management system to support adults with type 2 diabetes. The system comprises of a mobile app, automated text-messaging feedback, and a clinician portal. Blood glucose level (BGL) data are automatically transferred by a Bluetooth-enabled glucose meter to the clinician portal via the mobile app. The outcome measures of the study are firstly, to improve glycemic control and secondly, to improve the patient experience, reduce reliance on physical clinics, and decrease service delivery costs.

## 3. Conclusions

As we have shown in the papers that we have included in this special issue, e-health covers a wide range of topics and methodologies. The services presented in this special issue show great promise, and some may have clear advantages compared to the current standard approaches that they might be either supplementing or replacing. 

However, while many e-health services show great promise in trial phases, their implementation into the health services has often proven to be more difficult. The adoption of e-health is challenging because it involves not only individual patients and clinicians, but also very large and complex organizations with a range of organizational, bureaucratical, and managerial components [29]. Moreover, the implementation of new e-health services is often not sufficiently financially incentivized—an important issue that needs to be addressed by policy-makers that aim to encourage e-health use. The relationship between providers and patients is a cornerstone of health care [30,31,32], and e-health services that are able to integrate this perspective could be more likely to stand the test of time.

Despite these challenges, we are convinced that as technological development progresses, we are likely to see the testing and widespread implementation of increasingly more advanced e-health services that will help to provide better care for patients and reduce the strain on traditional services and costs to individuals and society.
